# The Outcomes of Isolated Tibiocalcaneal Arthrodesis: A Systematic Review

**DOI:** 10.1177/24730114241247547

**Published:** 2024-05-07

**Authors:** Charlotte Cossins, Ben George, Adrian J. Talia, Constantinos Loizou, Adrian Kendal

**Affiliations:** 1 Nuffield Orthopaedic Centre, Oxford, United Kingdom; 2 Department of Orthopaedic Surgery, Western Health, Footscray Hospital, Footscray, Australia; 3 The Nuffield Department of Orthopaedics, Rheumatology and Musculoskeletal Sciences, Oxford, United Kingdom

**Keywords:** tibiocalcaneal, fusion, arthrodesis, hindfoot, ankle, isolated

## Abstract

**Background:**

Tibiocalcaneal arthrodesis (TCA) can be achieved by internal fixation (intramedullary nail or plate), external fixation, or a combination. Evidence for the optimal approach is limited. This systematic review examines the outcomes of these different approaches to guide surgical management.

**Methods:**

A MEDLINE and Oxford SOLO search was performed using “tibiocalcaneal,” “ankle,” “fusion OR arthrodesis.” The primary outcome was union. Secondary outcomes included rates of postoperative complications, weightbearing status, rates of revision surgery, and PROMs. We included any studies with follow-up greater than 6 months that contained our primary outcome and at least 1 secondary outcome.

**Results:**

The initial search yielded 164 articles, of which 9 studies totaling 53 cases met the criteria. The majority of articles were excluded because they were nonsurgical studies, or were not about isolated TCA but were for tibiotalocalcaneal arthrodesis, more complex reconstructions (eg, Charcot), case reports, and/or did not include the predetermined outcome measures.

TCA union rate was 86.2% following external fixation, 82.4% for intramedullary nail fixation, and 83.3% for plate fixation. One patient underwent a hybrid of external and internal fixation, and the outcome was nonunion. The rate of complications following TCA was 69.8%.

**Conclusion:**

There is limited evidence on the best operative approach for isolated tibiocalcaneal arthrodesis. Both external and internal fixation methods had comparable union rates. External fixation had frequent complications and a more challenging postoperative protocol. Novel techniques such as 3D-printed cages and talus replacement may become a promising alternative but require further investigation.

## Introduction

Isolated talar pathology with catastrophic bone loss is commonly caused by traumatic avascular necrosis, infection, or post total ankle replacement and remains a surgical challenge.^20,23^ Treatment options include limb preservation techniques (such as ankle arthrodesis with bone grafting or custom total ankle replacement) or a below-knee amputation. Direct tibiocalcaneal arthrodesis (TCA) is a salvage procedure with fusion of the tibia to the calcaneus, aiming to create a stable plantigrade foot.^22^ This can relieve pain, correct malalignment, and enable patients to regain functional independence.

TCA can be achieved by internal (IF) or external fixation (EF), or a combination of both. A successful operation will restore anatomical alignment, achieve maximal contact between the tibia and calcaneus with minimal soft tissue and vascular disruption, and promote union through stable fixation.^25^ Reported IF techniques include either intramedullary nail (IF-N) or plate (IF-P) fixation. Ilizarov or fine-wire frame fixation has been used in EF and has been reported alone or in combination with IF.^1^ Nonunion, infection, hardware failure, and subsequent below-knee amputation are the main complications of all TCA and occur in all described fixation techniques.^15^

Currently, there is no clear evidence regarding the optimal fixation strategy in TCA. In addition, evaluating the literature on this area is challenging as many studies typically include TCA as part of combined procedures to treat Charcot patients with complex foot and ankle deformity, rather than isolated TCA. This systematic review evaluates the union rates of different surgical strategies for isolated TCA.

## Methods

A systematic review of the literature concerning isolated TCA was performed. For the purposes of this review, isolated TCA was defined as any surgical procedure in which the distal tibia was prepared, reduced, and fixed against the prepared surface of the calcaneus to achieve bone union.

### Outcome Measures

The primary outcome measure was rate of isolated tibiocalcaneal union. Secondary outcome measures include rates of superficial and deep infection, other postoperative complications, weightbearing status, rates of revision surgery, and patient-related outcome measures (PROMs).

### Search Strategy

**Figure 1.**
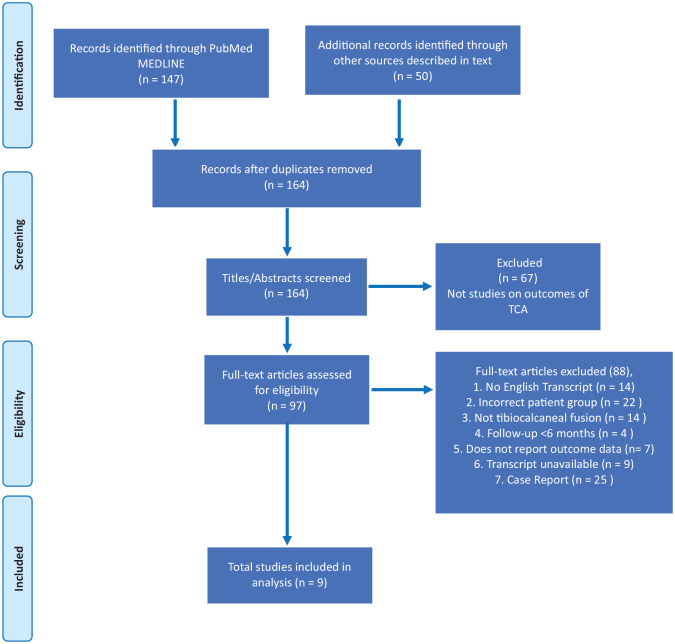
Preferred Reporting Items for Systematic Reviews and Meta-Analyses (PRISMA) flowchart outlining the process of study inclusion/exclusion according to the PRIMSA protocol.^19^

The Preferred Reporting Items for Systematic Reviews and Meta-Analyses (PRISMA) 2020 checklist was followed (see Figure 1).^19^ The explicit overarching question for the review was defined as “what are the outcomes of TCA?”

The systematic search was performed on PubMed and Oxford SOLO (providing additional access to Embase, Medline, ScienceDirect, SCOPUS databases) from inception to February 1, 2023.

To sample as broadly as possible, the following search terms were used: “tibiocalcaneal,” “ankle,” “fusion” AND/OR “arthrodesis.” All articles written in, or translated into, English were included. Search filters included primary clinical trial, meta-analysis, randomized controlled trial, review, and systematic review. Two authors independently completed title, abstract, and full-text screening. These broad criteria yielded more than 200 articles, of which 67 were immediately rejected because they were not TCA studies.

### Inclusion Criteria

Selection of the remaining studies was based on inclusion and exclusion criteria that were predetermined using the Population, Intervention, Comparison, Outcome, Study model (detailed below and Figure 1). Articles were included if the manuscript was written in English and reported surgical approaches and outcomes for isolated TCA in adults. All methods of internal and external fixation were included.

Articles that reported data on the primary outcome measure (rate of TCA union) and at least one of the prestated secondary outcome measures were included. We included case series with a minimum of 3 patients, cohort studies and RCTs. Where possible, we extracted data on individual participants included in wider, mixed cohorts. For example, we extracted data from case series that included patients with neuropathic ankle pathology and others with isolated talar pathology, so long as the data on isolated TCA was clearly separate and included the predetermined outcome measures.

### Exclusion Criteria

The review did not include articles on tibiotalocalcaneal fusion, triple hindfoot fusion, or pan-talar fusion procedures. “Blair fusions” in which a sliding graft from the distal, anterior surface of the tibia is placed into the residual talus (but the subtalar joint remains spared) were also excluded. Articles were excluded if TCA was performed as part of a more complex surgical procedure to correct foot deformity, most commonly for Charcot arthropathy. Studies involving a pediatric population were excluded. Any study that did not include bony union as an outcome measure had less than 6 months’ follow-up data and/or included single case reports was excluded.

### Data Extraction

Data extraction was performed by authors B.G. and C.C.

## Results

A total of 164 articles were identified from databases searching for title and abstract screening using the terms “tibiocalcaneal,” “ankle,” and “fusion OR arthrodesis.” Nine met the inclusion criteria (Figure 1). Six of these were retrospective case series that included patients who either had surgery to the ankle other than TCA,^18,31^ or had a mixed cohort of trauma and neuropathic ankle patients.^7,17,26,29^ In these broader studies, we extracted specific patients who met our inclusion criteria (see Figure 1).^19^

### Study Characteristics and Patient Demographics

Fifty-three cases of TCA on 53 patients were included. The mean age was 50.2 years (SD = 15.4). Thirty-three cases were male and 19 were female. One study did not report individual participants’ sex (Table 1).^26^

**Table 1.** A Summary of Study Characteristics and Patient Demographics Including Comorbidities and Smoking Status.

Study	N	Mean Age	Male:Female	CAD/HTN	Smoking	Diabetes	Follow-up, mo (range)	MSK Comorbidity (n)
Rochman et al^23^ (2008)	11	44.0 (29-77)	10:1	N/A	N/A	N/A	35 (12.5-81.5)	N/A
Dennison et al^6^ (2001)	6	44.5 (27-67)	2:4	N/A	N/A	N/A	32 (13-49)	N/A
Hofmann et al^10^ (2009)	9	55.0 (36-75)	6:3	4	N/A	1	12	6
Ordas**-**Bayon et al^18^ (2021)	10	48.9 (22-72)	7:3	2	1	0	23.2 (12-56)	0
Zarutsky et al^31^ (2005)	3	45.7 (37-49)	1:2	0	0	0	27	0
Myerson et al^17^ (2000)	4	63.3 (47-72)	1:3	N/A	N/A	0	48 (19-112)	2
Smith et al^26^ (2017)	1	42	0:1	N/A	1	0	17.5 (±5.3) ^a^	N/A
Gorman et al^7^ (2016)	1	56	1:0	N/A	0	0	47 (±28)^a^	N/A
Vitiello et al^29^ (2020)	8	55.6 (27-76)	4:4	2	N/A	8	55.4 (27-121)	N/A

Abbreviations: CAD, presence of coronary artery disease; HTN, presence of hypertension; MSK, musculoskeletal; N/A, not detailed in the study.

a If unable to determine follow-up for cases extracted from a larger cohort, the mean follow-up for the whole cohort is reported and indicated here.

The majority, 40 cases (75.5%), were a result of talar trauma.^6,10,17,18,23,26,29,31^ The time from traumatic event to TCA varied between cases: 2 were performed acutely,^18^ 2 occurred within 2 weeks post debridement and initial stabilization of the joint,^23^ 2 occurred 4 and 6 weeks postinjury, because of management of soft tissue injury with flaps.^18^ Fifteen TCAs were performed following infection post ankle surgery for a variety of traumatic injuries.^10,18,23,31^ The time from event to TCA was only reported for 8 of these patients, and the average was 30 weeks (range: 2-104).^10,18^

Trauma-related avascular necrosis accounted for 8 cases.^6,17,18^ Time to TCA was reported for 7 patients, and on average was 192 weeks (3-924 weeks).^6,18^ One case had talar necrosis 27 years after the initial injury, requiring TCA.^10^

TCA was indicated in 3 cases owing to arthritis secondary to trauma,^10,26,29^ including 1 case of avascular necrosis.^10^ No information was given about the date of the initial traumatic event. Three cases of TCA were due to nonunion of previous ankle surgery. One case was performed 9 months after the initial injury, with the 2 other cases occurring several years later.^23,29^ For 4 cases, there is no information on the time or indication of TCA other than trauma.^10,23,31^

Four cases were due to talar osteonecrosis, with no cause reported.^29^ Inflammatory arthritis-related accounted for 5 cases.^17,29^ Tibiocalcaneal arthritis following talectomy was reported in 1 case.^7^ Chronic osteomyelitis accounted for 1 case.^10^ In 1 case, the indication was arthritis, with no cause of arthritis noted.^10^

Five studies reported on patient comorbidities,^10,17,18,29,31^ with only 4 reporting on smoking status.^7,18,26,31^ One-fourth (26.7%) of patients had cardiovascular disease, and 13.3% of patients had a smoking history. Seven studies reported on the diabetes status of patients, and 25.0% of patients had diabetes. Five articles reported on body mass index,^7,10,18,26,31^ while only being reported for individual participants in 4 of these. More than half (53.3%) of these participants were obese (body mass index >30) (Table 1).

### Intraoperative details

The average number of operations prior to TCA was 2.5. Eighteen patients had at least 1 operation, of which 1 had 2 operations, 5 had 3 operations, 6 had 4 operations, and 6 had more than 5 operations (Table 2).

**Table 2.** Operative Details Broken Down by Article.^a^

Reference	Viable Talar Head	Method of Fixation	Bone Graft	Average no. of Previous Operations (Range)	Preoperative Infection	Leg Lengthening
Rochman et al^23^ (2008)	4 nonviable 7 viable	Ilizarov external fixator	No	1.27 (0-3)	Deep infection (n = 7)	8 cases
Dennison et al^6^ (2001)	Preserved talar head where possible	Ilizarov external fixator	No	5.00 (1-16)	0	8 cases
Hofmann et al^10^ (2009)	8 viable talar head 1 nonviable talar head	Retrograde intramedullary nail	Yes, from iliac crest or lateral malleolus	5.67 (0-20)	Deep infection (n=5)	0 cases
Ordas**-**Bayon et al^18^ (2021)	Preserved talar head where possible	Taylor Spatial Frame ± hindfoot fusion intramedullary nail	Yes (2 patients)	3 (0-8)	Deep infection (n = 4)	4 cases
Zarutsky et al^31^ (2005)	No	External fixator (circular)	Yes	Not reported	Deep infection (n = 1)	0 cases
Myerson et al^17^ (2000)	Preserved talar head where possible	Lateral approach condylar blade plate	Yes, fibular	0.25 (0-1)	Superficial infection (n = 2)	0 cases
Smith et al^26^ (2017)	No	Lateral approach locking plate	Yes, fibular	0	0	0 cases
Gorman et al^7^ (2016)	No	Posterior blade plate	Yes, morselized bone from posterior tibia	1	0	0 cases
Vitiello et al^29^ (2020)	N/A	Retrograde Intramedullary nail	No	0	0	0 cases

Abbreviation: TCA, tibiocalcaneal arthrodesis.

a These include the specific surgical approach; the usage of bone graft; number of surgical interventions prior to TCA; the proportion of cases where the talar head was viable; the presence of infection in the limb immediately before the operation was performed; and the number of cases involving simultaneous leg-lengthening.

EF was used in 29 cases of TCA,^6,23,31^ of which 17 used the Ilizarov frame,^6,23^ 2 used an undefined circular external fixator,^31^ and 10 used a Taylor Spatial Frame.^18^ In 1 case, EF was combined with IF; however, the method of IF is not described.^31^ TCA was fixed with plating systems in 6 patients,^7,17,26^ and an intramedullary nail alone in 17 patients.^10,29^

In 5 studies, a subgroup had preservation of the talar head.^6,10,17,18,23^ In these studies, it was apposed to the distal tibia. If the talus was completely excised, the tibia was apposed to the navicular. For 1 patient, additional screws were placed at the tibionavicular and calcaneocuboid joints.^10^

Morselized bone graft was used to augment TCA in 17 patients across 6 studies.^7,10,17,18,26,31^ This included autologous graft from the iliac crest or lateral malleolus in 9 cases,^10^ from the iliac crest or lateral malleolus in combination with demineralized bone matrix and platelet-derived growth factors in 2,^18^ from the fibular head in 5,^17,26^ and the posterior tibia in 1.^7^

Where infection was present, management included debridement and insertion of antibiotic beads or pellets, alongside parenteral antibiotics.

Simultaneous TCA and leg-lengthening via bone transport at the proximal tibia was performed in 22 patients with EF.^6,18,23^ Patients were kept nonweightbearing during bone transport and had periodic radiographs to assess bone lengthening. The remaining 7 cases did not undergo leg-lengthening as they were assessed as unable to tolerate bone transport.

### Primary Outcome

The primary outcome measure (union) is summarized in Table 3. The mean TCA union rate was 83% (44 of 53). For EF, this was 86.2% (25 of 29).^6,18,23,31^ IF-N had a mean union rate of 82.4% (14 of 17).^10,29^ IF-P had a union rate of 83.3% (5 of 6).^7,17,26^

**Table 3.** A Summary of Union Rates Achieved With Each Method of Fixation.

	Cases	Union	Rate, %	Studies
External fixation	29	25	86.2	4 (Rochman et al^23^, Dennison et al^6^, Zarutsky et al^31^, Ordas-Bayon et al^18^)
Internal fixation (nail)	17	14	82.4	2 (Hofmann et al^10^, Vitiello et al^29^)
Internal fixation (plate)	6	5	83.3	3 (Myerson et al^17^, Smith et al^26^, Gorman et al^7^)
Combined internal and external fixation	1	0	0.0	1 (Zarutsky et al^31^)

There were 9 cases of nonunion. One patient died before union could be established. One patient had nonunion following a hybrid of external and internal fixation. In 2 cases, nonunions were associated with IF infection and were managed with removal of hardware, debridement, and antibiotics. Two cases had IF with an intramedullary nail following nonunion with EF. Two cases had no further surgery owing to stable pseudoarthrosis, and subsequent treatment for 2 cases was not described.

#### Secondary outcomes

##### Postoperative complications

The average complication rate, including major and minor complications, was 69.8% (37 of 53) (Table 4).

**Table 4.** Secondary Outcomes Measures Including Postoperative Complications (*x/n* Where *x* = Number of Complications and *n* = Number of Cases).^a^

	Superficial Infection	Deep Infection	Revision Surgery	Time to Full Weightbearing	PROMs	Studies
External fixation	27/29	0/29	8/29	Rochman et al^23^: 4-6 wk	AOS, 10-77 (38.6) Ordas-Bayon et al^18^ Kitaoka/Ptazer; poor to excellent Dennison et al^6^ AOFAS, 44-77 (65) Rochman et al^23^	Rochman et al^23^, Dennison et al^6^, Zarutsky et al^31^, Ordas-Bayon et al^18^
Internal fixation with nail	1/17	4/17	3/17	Vitiello et al^29^: > 50 d Hofmann et al^10^: 4- 20 wk	AOFAS; preop 32.1, postop 72.5 Hofman et al AOFAS; preop 40.9, postop 70.9 Vitiello et al^29^	Hofmann et al^10^, Vitiello et al^29^
Internal fixation with plate	1/6	1/6	No data	Gorman et al^7^: 6-10 wk	No data	Myerson et al^17^, Smith et al^26^, Gorman et al^7^
Combined external and internal fixation	No data	No data	No data	No data	No data	Zarutsky et al^31^

Abbreviations: AOFAS, American Orthopaedic Foot & Ankle Society ankle-hindfoot score; PROMs, patient-related outcome measures.

a PROMs (scoring system, range [mean]) and time to full weightbearing are reported for each article that included them.

In the EF group, 93% of the cases (27 of 29) were associated with 1 or more complications. There were 27 pin site infections. One case reported anaphylaxis secondary to antibiotics. There were no cases of new deep infection. In 2 cases, there were problems with the proximal wires of the frame, 1 of which required a general anesthetic to resite the wires. There was 1 case of malunion. There was 1 case of delayed bony healing that required bone stimulator. There was 1 case of wound slough that delayed wound healing. There was 1 case of chronic pain secondary to reflex sympathetic dystrophy. There were complications in 3 cases related to the leg-lengthening component of the operation.

In the IF-N group, 41.2% of cases (7/17) were associated with 1 or more complications. There was 1 case of wound dehiscence, which was treated with antibiotics. There were 2 cases of new deep infection that required hardware removal and washout. In 1 case, they died 8 weeks postoperatively from a PE. There were 2 cases of ongoing pain. There was 1 case of lymphedema. One case was related to the loss of a gracilis flap that was used to correct a soft tissue defect that was associated with the injury.

In the IF-P group, 50% of cases (3 of 6) had 1 or more complications. There was 1 case of new deep infection, 1 case of cellulitis, and 1 case of talar head necrosis.

##### Resolution of preexisting infection

Seventeen cases had preexisting joint infection at the time of surgery (Table 2). Infection resolved in 15 cases. Twelve of these cases were managed with EF, and 3 were managed with IF-N.^10,18,23,31^ Chronic osteomyelitis in 2 IF-N cases persisted postsurgery.^10^

##### Revision surgery

Overall, 3 patients underwent revision post IF-N (all deep infection) and 8 EF patients required revision surgery (3 for nonunion, 3 for leg length discrepancy, and 2 due to malunion). No IF-P patients were reported to have revision surgery.

##### Time to weightbearing

Six studies reported time to weightbearing.^7,10,17,23,29,31^ For IF-P, patients were nonweightbearing for 3 months, then in a weightbearing cast for 2-3 months, and then an AFO or shoe as tolerated.^17^ Following IF-N, the average time to fully weightbearing was a minimum of 50 days.^10,29^ It took 2-6 weeks for patients treated with EF to be fully weightbearing.^23,31^ The mean time of frame treatment was 8.94 months (5-13 months).

##### Patient-reported outcome measures

There was heterogeneity in the PROMs used. Three studies used AOFAS,^10,23,29^ 1 used AOS and SF-36,^18^ and 1 used the Kitaoka and Ptazer classification.^6^ One study did not report either pre- or postoperative PROMs.^31^ Two IF-N studies reported both preoperative and postoperative PROMs, both showing a significant improvement in mean AOFAS score (Table 4).^10,29^

## Discussion

The systematic review evaluated current literature regarding union of isolated TCA as a salvage procedure for significant talar bone loss. To our knowledge, this is the only review reporting on different surgical approaches for isolated TCA.

The overall union rate of TCA was 83.6% and is similar to rates reported for tibiotalocalcaneal arthrodesis (TTCA).^16^ The majority of patients underwent EF rather than IF. This may represent concerns over the surrounding soft tissue envelope following trauma and the additional advantages in infection. It can also be combined with bone transport and used to address LLD.^23^ However, EF requires more resource-intensive postoperative management.^28^ The postoperative protocols are complex particularly when simultaneous leg-lengthening is performed.^28^ Pin site infections are common and need treating early to prevent deeper infections.^8^ Patients also require a further hospital admission to remove the frame when union is achieved. Nonetheless, it remains an option for isolated TCA in complex, comorbid patients, particularly in cases of infection, and had the highest union rate in this review (86.2%).

Kugan et al^14^ published a case series of 48 patients with complex ankle/hindfoot fusions. This population did not meet the inclusion criteria for our review; however, the results prove a useful comparison to our own data. In their population, EF achieved successful union in 32 of 36 patients (88.9%) who had TTCA vs 8 of 12 (66.7%) patients who had TCA, further illustrating the inherent challenges of TCA. IF-N was used to revise 7 of the 8 nonunions, of which 5 then united. Similarly, a nail was used to revise 2 TCA nonunions in a series of 10 EFs.^18^

In a 386-case systematic review of TTCA, there was no statistically significant difference in union rate when comparing IF-N vs IF-P fixation.^9^ However, there was a significantly higher complication rate associated with IF-P (30%) compared with the IF-N (20%). Similarly, in our review IF-P was associated with a higher complication rate (50%) than IF-N (41.2%).

A recent study concluded that obesity is not significantly associated with poorer post-ankle arthrodesis outcomes, but that morbid obesity is.^13^ In our analysis, the rate of nonunion in obese participants equaled those with a body mass index <30. This is likely to reflect the scarcity of comorbidity data. Similarly, we found no correlation with smoking and nonunion in TCA despite evidence that it is associated with a 3.75 times increase in nonunion rate following ankle arthrodesis when adjusting for other confounding factors.^3^ It has recently been shown to be an independent risk factor for nonunion following arthroscopic ankle fusion.^30^

Because of the rarity of isolated TCA, there are few studies available for data extraction and analysis. The small sample sizes and the lack of control groups in the studies reviewed were major limitations. Outcome measures were heterogeneous, especially for patient-reported outcomes. Only 1 of the studies reported preoperative PROMs.^10^ Larger sample sizes are required to enable informative statistical analysis and therefore to provide more clarity regarding outcomes.

In TCA, the excised talus leaves a ≥3-cm LLD.^22^ Three of the 4 EF articles used simultaneous lengthening to address the LLD.^6,18,23^ The remaining articles used some form of morselized bone grafting to fill smaller defects or aid union.^10,17,26^ In 1 case, a combination of lengthening and graft implantation was used.^18^ As previously mentioned, cases where LLD was significant, heel rise orthotics can be used.^6,18^

Two recent studies reported union rates with femoral head allografts of 88%^24^ and 89%.^4^ A promising alternative to the graft is the use of a 3D-printed cage, often used in conjunction with intramedullary nailing. It allows complete excision of the necrotic talus and avoid the difficulties associated with EF and lengthening while providing a more stable weight bearing structure than bone graft alone would.^5,11^ A larger study of 21 patients who underwent ankle fusion with a custom cage was associated with a union rate of 95.2% and failure in 2 cases (9.5%).^21^ PROMs revealed a significant improvement in VAS pain score (decrease of 59.3/100) alongside a more modest improvement in functional scores (Foot and Ankle Ability Measure activities of daily living subscale and the 12-Item Short Form Health Survey). However, there is a cost barrier to custom implants and little data on cost-benefit analyses. Steele and colleagues^27^ compared the outcomes of TTCA when using a femoral head allograft to the use of a 3D-printed implant. The use of femoral head allograft was associated with a low success rate of 42.9% (defined as union across all 3 articulations). This was compared to a rate of 75% with a 3D-printed spherical implant. The group reported that graft resorption occurred in 57.1% of the femoral head group, contributing significantly to the union failure.

Total talar replacement (TTR) is another alternative to addressing talar destruction. It reconstructs the ankle-hindfoot complex and preserves mobility, a considerable advantage over the arthrodesis procedures.^12^ Results from a 27-patient case series showed that TTR was associated with statistically significant improvements in PROMs. These results are limited by lack of long-term follow-up as this is an area of ongoing research. A large systematic review (161 cases) of outcomes following TTR demonstrated significant improvement in PROMs with mean AOFOS (American Orthopaedic Foot & Ankle Society Score) preoperatively 27.93 to 81.99 postoperation. There were comparatively low complication rates, with only 1 of 161 requiring amputation and an overall complication rate of 9.32% (15 of 161) after a mean follow-up of 37.35 months.^2^ However, as with 3D-printed cage implants, TTRs are costly and with, currently, limited supporting evidence.

## Conclusion

Isolated TCA remains an option for patients with total or near total talar loss, most commonly due to trauma, avascular necrosis or infection. All 3 approaches (external fixation, plate and nail internal fixation) result in comparable union rates. In all studies, the weightbearing status was improved following the operation. However, the postoperative complication rate was high (66.6%). The few and heterogenous cases extracted from small studies that rarely include comorbidity data or PROMs limit the validity or reproducibility of these conclusions.

## Supplemental Material

sj-pdf-1-fao-10.1177_24730114241247547 – Supplemental material for The Outcomes of Isolated Tibiocalcaneal Arthrodesis: A Systematic Review Supplemental material, sj-pdf-1-fao-10.1177_24730114241247547 for The Outcomes of Isolated Tibiocalcaneal Arthrodesis: A Systematic Review by Charlotte Cossins, Ben George, Adrian J. Talia, Constantinos Loizou and Adrian Kendal in Foot & Ankle Orthopaedics
